# The College of Nursing: Wanted Potential Energy!

**Published:** 1919-09-06

**Authors:** 


### THE COLLEGE OF NURSING: WANTED POTENTIAL ENERGY!

Hercules in a Lift.

Has the trained nurse, actual or prospective member of the College of Nursing, considered the term potential energy? This is supposed to be the silly season, and the question may seem to correspond. Potential energy is not by any means a sweetmeat, nor the sort of thing that one likes to contemplate by way of entertainment at the seaside. I am not an authority on the subject. I happened to catch the expression in passing, and, somehow, it haunts. In reality I think it is one of those catch-words which men of science avail of in order to maintain that atmosphere of exclusiveness which seems to surround their particular domain.

I had occasion recently to visit a friend in the City. His office is on the fifth floor, and as I am not partial to stone steps I ascended in the lift. I gave the usual signal and in a moment the door opened, and I found myself in the custody of a giant. What a man! He stood at least six feet four, and in his uniform he seemed to be at least half as broad as he was long, and as deep as he was broad—an ideal model of Hercules. And there he was, pressing a button. He did it all right. I took my stand beside the giant, humble in soul, and duly impressed with a sense of my own insignificance. When we reached the fifth floor the great man ushered me out with the air of a monarch. He seemed to me an ideal object-lesson for the Prime Minister on the subject of waste.

What has all this to do with the trained nurse and the College of Nursing? The question was inevitable, and I was expecting it. I venture to think—quite a lot. The College consists of some fourteen or fifteen thousand members and an Executive Committee. Without pausing to discuss what the College has accomplished, or is prepared to accomplish, the paramount feature of the situation is that fifteen thousand intelligences are, so far as the College is concerned, unemployed. An army of such dimensions if properly directed and wholly active might move mountains. There is, indeed, no limit to the possibilities of their achievement. I do not think I exaggerate when I say that at present they wait *en masse* for the Executive to work out their salvation and their emancipation. This is a policy which accords with pre-war conceptions. But in the five tragic years that have passed we have lived through æons of time, and we must cast our horoscope accordingly. The gospel of the day is that latent energy must be used, and not left quiescent. Let me illustrate. The Government has recently issued an edict requiring the heads of Civil Departments of State to form councils in every public office consisting of representatives of the official side and of the staff side, with the dual object of promoting efficiency and economy, and also, be it noted, of benefiting the employees. In the future the staff is requested to suggest better methods of working, to discuss principles of promotion, and at the same time to put forward schemes calculated to result in their own betterment. The new regime sounds almost Bolshevik as compared with pre-war ideals, when men were pawns and when the doctrine of circumlocution and delay reigned supreme. Government officials used to be compared with the fountains in Trafalgar Square, which played from ten to four. Now they are asked to co-operate in promoting retrenchment and reform, and in the transition they are to benefit. I mention this merely to illustrate the spirit of the time. In the most conservative quarters it is recognised that the worker is to be the saviour of the country. It is no longer a problem of keeping him in his place, but of soliciting his co-operation in great schemes of national reform.

I am thinking of the fifteen thousand who belong to the College of Nursing. What of them? They are, no doubt, pursuing their avocations, but, so far as the College of Nursing is concerned, they are chiefly idle. And they possess tons of intelligence. Acting jointly and severally they could, if properly directed and inspired, as I have said, move mountains. Members of Parliament, hospital committees, and all the other official embroideries are trifles light as air compared with the potential energy which such a corporation holds in the hollow of its hand.

I am driven to the conclusion that the College Executive is slumbering. A superb machine has been constructed. The College has within its own programme the power to gather together in one fold the entire nursing strength of the Kingdom, nay, of the Empire, and, instead of supplicating members of Parliament, dictating to them. The radical mistake that Mrs. Fenwick and her faction have made for thirty years is in continually petitioning Parliament to do what nurses themselves can do a thousandfold better. Herein, I hold, lies the degradation of the nurse. I am a complete believer in the College of Nursing so far as its programme is concerned. Much has been accomplished, but a vast deal remains, and the whole fifteen thousand stand to attention, awaiting direction. Hercules is there, but his duties consist of pressing a button.

